# Distribution of £110,000

**Published:** 1920-08-07

**Authors:** 


### METROPOLITAN HOSPITAL SUNDAY FUND.

### Distribution of £110,000.

The Council of the Metropolitan Hospital Sunday Fund met on August 5 to receive the report of the Distribution Committee and to sanction the awards recommended. A report of the proceedings will be published in our next issue. Meanwhile we have the pleasure to give the Distribution Committee's report and the list of awards, which amount to no less a sum than £110,000, which is £25,000 more than the largest amount hitherto distributed by the Sunday Fund in any one year.

### ANNUAL REPORT OF THE COMMITTEE OF DISTRIBUTION.

The Committee of Distribution held their first meeting on Friday, 11th June, and then, and at subsequent meetings, examined the reports and income and expenditure accounts of the several institutions seeking to participate, and made their awards in conformity with the Laws of the Constitution.

The Committee beg to report that the total of the Fund on August 7 will amount to £111,282.

This year 220 institutions have applied to participate in the Fund, being eight less than in 1919, and the Committee now recommend the distribution of £110,000 to the several hospitals, institutions, dispensaries, and nursing associations, shown in the appendix hereto.

The Committee having reduced or postponed the determination of the bases of award to several institutions, the governing bodies of the same were informed thereof in accordance with the Laws of the Constitution. Seven deputations attended the Committee, and, after hearing their several explanations, and those of the remainder having been received by letter, the bases of award were finally determined.

The Committee have felt obliged to recommend awards on a somewhat reduced basis to those of the cottage hospitals, where the low daily average occupation of beds is responsible for an unreasonably high cost per in-patient per week.

The Committee held a special meeting on July 6, 1920, to consider whether, in the interests of the hospitals, it was desirable that the Hospital Sunday Fund should take similar action to that of King Edward's Hospital Fund for London by using some of its capital for increasing the grants made to the hospitals and dispensaries this year. The Committee decided not to recommend such action to the Council, leaving it to the Council to take such a step if thought advisable.

Seven and a-half per cent. of the total sum available for distribution is appropriated to the purchase of surgical appliances during the ensuing year, and 2½ per cent. for district nursing associations.

In compliance with an order of the Council, statistics are being prepared as usual for the use of its members, showing the number of beds in hospitals, the cost of patients at hospitals and dispensaries, the proportionate expense of management to maintenance, and other information. A copy thereof will be sent to each member of the Council.

Edward E. Cooper, Lord Mayor, President and Treasurer; R. Holland-Martin, Vice-President; William S. Church, Chairman of Committee; W. Hardy Harwood, Somerleyton, John C. Bell, Leonard L. Cohen, G. H. Makins, Adrian Pollock, Marcus Samuel, W. Hale White.

Hon. Secs. { C. C. Wakefield, John Henry Buxton,

The Mansion House, August 1920.

### THE AWARDS RECOMMENDED.

#### THIRTY-SIX GENERAL HOSPITALS.

Charing Cross Hospital, £2,350; French Hospital, £250; German Hospital, £350; Great Northern Central Hospital, £2,800; Guy's Hospital, £4,800; Hampstead and N.W. London Hospital, £1,450; Italian Hospital, £100; Kensington General Hospital, £250; King's College Hospital, £3,950; King Edward's, Ealing, £250; London Hospital, £12,000; London Homœopathic Hospital, £1,100; London Temperance Hospital, £1,000; Metropolitan Hospital, £1,850; Middlesex Hospital and Convalescent Home, £3,750; Mildmay Hospital, £400; Mildmay Memorial Hospital, £125; Miller Hospital and Royal Kent Dispensary, £1,150; Phillips' Memorial Homœopathic Hospital, £75; Poplar Hospital, £800; Prince of Wales' Hospital, Tottenham, £1,500; Royal Free Hospital, £2,950; St. Andrews', Dollis Hill, £100; St. George's Hospital, £4,150; SS. John and Elizabeth Hospital, £375; St. John's Hospital, Lewisham, £350; St. Mary's Hospital, £2,850; St. Thomas's Hospital, £3,600; Seamen's Hospital Society, £1,550; University College Hospital, £2,550; Walthamstow, etc., Hospital, £260; Wandsworth, Bolingbroke Hospital, £700; West Ham, Queen Mary's, £1,750; West London Hospital, £2,550; Westminster Hospital, £2,300; Wimbledon Hospital, £150.

#### SIX CHEST HOSPITALS.

City of London Hospital for Diseases of the Chest, Victoria Park, £1,750; Hospital for Consumption, Brompton, £2,450; Mount Vernon Consumption Hospital, £500; Royal Hospital for Diseases of the Chest, £450; Royal National Sanatorium, £50; Royal National Hospital, Ventnor, £400.

#### TWENTY CHILDREN'S HOSPITALS.

Alexandra Hospital for Hip Disease, £150; Banstead Surgical Home, £50; Barnet Home Hospital, £40; Belgrave Hospital, £510; Cheyne Hospital for Incurable Children, £275; East London Hospital for Children, £1,250; Evelina Hospital for Sick Children, £500; Home for Incurable Children, £100; South-Eastern Hospital for Children, Sydenham, £375; Hospital for Sick Children, £2,050; Infants' Hospital, Westminster, £450; Kensington, for Children, and Dispensary, £150; Paddington Green Hospital for Children, £700; Queen's Hospital for Children, £2,350; Royal Waterloo Hospital for Children and Women, £700; St. Mary's Hospital, Plaistow, £475; St. Monica's Hospital, Brondesbury, £120; Victoria Hospital for Children, Chelsea, £950; Victoria Home, Margate, £40; Hospital for Hip Disease, Sevenoaks, £50.

#### NINE LYING-IN HOSPITALS.

British Home for Mothers and Babies, £50; City of London Maternity Hospital, £425; Clapham Maternity Hospital and Dispensary, £15; East End Mothers' Home, £200; General Lying-in Hospital, £525; Jewish Maternity, £120; Plaistow Maternity Hospital, £125; Queen Charlotte's Lying-in Hospital, £1,000; Salvation Army Lying-in Home, £75.

#### SIX HOSPITALS FOR WOMEN.

Chelsea Hospital for Women, £450; Hospital for Women, Soho, £650; Grosvenor Hospital for Women, £250; Garrett Anderson Hospital, £900; Samaritan Free Hospital, £900; South London Hospital, £575.

#### TWENTY-ONE OTHER SPECIAL HOSPITALS.

Middlesex Hospital, Cancer Wing, £400; London Fever Hospital, Islington, £275; St. Mark's Hospital for Fistula, £600; National Hospital for the Diseases of the Heart, £250; Female Lock Hospital, £600; Male Lock Hospital, £200; Hospital for Epilepsy, Paralysis, etc., £525; National for Paralysis Hospital, £1,550; West End Nerves Hospital, £525; Central London Hospital, £375; Royal Eye Hospital, £325; Royal London Ophthalmic Hospital, £1,525; Royal Westminster Hospital, £300; Western Hospital, £200; Royal National Orthopædic Hospital, £700; Royal Sea-Bathing Hospital, £125; St. John's Skin Hospital, £100; St. Peter's Stone Hospital, £350; Central London Throat Hospital, £125; Throat Hospital, Golden Square, £200; Royal Dental Hospital, £225.

**Metropolitan Hospital Sunday Fund**—(*continued*).

TWENTY-SIX CONVALESCENT HOMES.

Metropolitan Convalescent Institution, Walton, £525; Metropolitan Convalescent Institution, Broadstairs, £150; Metropolitan Convalescent Institution, Bexhill, £900; All Saints' Convalescent Hospital, Eastbourne, £100; All Saints' Convalescent Home, St. Leonards, £30; Ascot Priory Convalescent Home, £15; Brentwood Convalescent Home for Children, £25; Catherine Gladstone, £50; Chelsea Hospital for Women, Convalescent Home, St. Leonards, £50; Hahnemann Convalescent Home, Bournemouth, £30; Fairlight, £75; Hemel Hempstead, £100; Herne Bay, Baldwin-Brown Convalescent Home, £75; Homœopathic Hospital Convalescent Home, Eastbourne, £20; Mary Yolland Home, £30; Police Seaside Home, Brighton, £110; St. Andrew's Convalescent Home, Clewer, £50; St. Andrew's Convalescent Home, Folkestone, £250; St. John's Home, Kemp Town, £100; St. Joseph's Convalescent Home, Bournemouth, £20; St. Leonards-on-Sea Convalescent Home, £200; Eversfield, St. Leonards, £50; St. Mary's Convalescent Home, Birchington, £50; St. Mary's Convalescent Home, Broadstairs, £200; St. Michael's Convalescent Home, Westgate, £20; Seaford Convalescent Home, £50.

NINETEEN COTTAGE HOSPITALS.

Acton Cottage Hospital, £175; Beckenham Cottage Hospital, £60; Blackheath and Charlton Cottage Hospital, £90; Brentwood Cottage Hospital, £45; Bromley, Kent, Cottage Hospital, £70; Canning Town Cottage Hospital, £140; East Ham Cottage Hospital, £50; Eltham Cottage Hospital, £80; Enfield Cottage Hospital, £150; Epsom and Ewell Cottage Hospital, £70; Hendon Cottage Hospital, £60; Hounslow Cottage Hospital, £80; Kingston Cottage Hospital, £90; Reigate and Redhill Hospital, £300; Sidcup Cottage Hospital, £40; Surbiton Cottage Hospital, £70; Tilbury (Passmore Edwards) Cottage Hospital, £80; Willesden Cottage Hospital, £100; Wimbledon (Nelson) Hospital, £175.

ELEVEN INSTITUTIONS.

Florence Nightingale Hospital for Invalid Gentlewomen, £300; St. Saviour's Hospital and Nursing Home, £150; Stoke Newington Home Hospital, £50; Firs Home, Bournemouth, £20; St. Columba's Hospital, £375; St. Luke's House, Pembridge Square, £150; Santa Claus Home, Highgate, £125; Royal Mineral Water Hospital, Bath, £50; Winifred House, Holloway, £75; Highgate Convalescent Home, £50; Holt Children's Sanatorium, £50.

THIRTY-FIVE DISPENSARIES.

Battersea Provident Dispensary, £140; Billingsgate Dispensary, £60; Brixton, etc., Dispensary, £60; Buxton Street Dispensary, £15; Camberwell Provident Dispensary, £40; Child's Hill Provident Dispensary, £6; City Dispensary, £70; Clapham General and Provident Dispensary, £25; Eastern Dispensary, £30; East Dulwich Provident Dispensary, £50; Farringdon General Dispensary, £40; Finsbury Dispensary, £70; Forest Hill Provident Dispensary, £35; Greenwich Provident Dispensary, £25; Hampstead Provident Dispensary, £45; Islington Dispensary, £50; Islington Medical Mission, £55; Kilburn, Maida Vale, and St. John's Wood Dispensary, £40; Kilburn Provident Medical Institution, £50; London Dispensary, Spitalfields, £20; London Medical Mission, Endell Street, £40; Margaret Street, for Consumption, £6; Metropolitan Dispensary, £30; Notting Hill Provident Dispensary, £20; Public Dispensary, £50; Queen Adelaide's Dispensary, £8; Royal General Dispensary, £30; St. George's, Hanover Square, Dispensary, £25; St. John's Wood Provident Dispensary, £35; St. Marylebone General Dispensary, £70; Stamford Hill, Stoke Newington Dispensary, £55; Western Dispensary, £55; Western General Dispensary, £80; Westminster General Dispensary, £40; St. Pancras, £80.

THIRTY-TWO NURSING ASSOCIATIONS.

Belvedere, Abbey Wood, £14; Brixton, £70; Central St. Pancras, £84; Charlton and Blackheath, £28; Chelsea and Pimlico, £70; Hackney, £56; Hammersmith, £70; Hampstead, £42; Isleworth, £28; Kensington, £140; Kilburn, £14; Kingston, £42; Lambeth Road (Catholic), £28; Metropolitan (Bloomsbury), £42; St. Olave's (Bermondsey), £42; Paddington and Marylebone, £126; Plaistow, £126; Ponders End, Enfield, etc., £28; Rotherhithe, £28; Shoreditch, £112; Sick Room Helps Society, £20; Sidcup, £14; Silvertown, £28; South London (Battersea), £126; Southwark, £70; South Wimbledon, £50; Tottenham, £50; Westminster, £56; Woolwich, £70; East London, £176; North London, £150; Ranyard Nurses, £750.

**Total awards 1920, £110,000.**

